# What determines the progress of online information access to banking corporate governance practices? The case of Paraguay

**DOI:** 10.1371/journal.pone.0262334

**Published:** 2022-01-05

**Authors:** Walter Daniel Ovelar-Fernández, María del Mar Gálvez-Rodríguez, Carmen Caba-Pérez

**Affiliations:** 1 Economics and Business Faculty, Autónoma de Asunción University, Asunción, Paraguay; 2 Economics and Business Faculty, University of Almeria, Almeria, Spain; University of Almería, SPAIN

## Abstract

This paper contributes to the lack of longitudinal studies concerning online information access to corporate governance (CG) practices in the banking sector of Latin American countries. In particular, this study aims to analyze the factors that influence information transparency, both mandatory and voluntary, related to CG practices of banks that operate in Paraguay via their websites from 2016 to 2019. Findings indicate the need to improve the level of information available on websites, with disclosure of voluntary information on CG practices being more prevalent than the disclosure of mandatory information. Likewise, banks that operate in Paraguay have made scant “progress” regarding online access to their governance information over the years analyzed. Moreover, the factors “Bank size” and “listed status” positively influence the information transparency regarding CG practices of Paraguayan banks. In contrast, “leverage,” “liquidity,” “size of the audit firm,” and “credit risk rating” are factors that have a negative relation with the extent of CG disclosure.

## Introduction

Information access or transparency on Corporate Governance (CG) practices is still a challenging issue for banks operating in Latin America (LA) since this region is characterized by opaque markets with high levels of information asymmetries, weak legal environments, and systematic corruption [1]. Consequently, providing relevant and credible information on the adoption of good governance practices is essential for establishing their legitimacy as valuable economic agents for national economic development and attracting national and international investors [2].

The literature regarding the transparency of CG practices of LA banks is scarce [3]. The existing studies mainly focus on non-financial companies and the disclosure of voluntary information through static analyses. In particular, these studies are developed in the context of Venezuela [4], Brazil [5–7], and Argentina [8]. An exception to this line of research is the work of Briano and Saavedra [9], who address several of the aforementioned LA countries along with Chile and México. The research on transparency in the banking sector focuses on information access to voluntary information from a general perspective [10]. Indeed, research on the CG transparency practices of LA banks is minimal [3].

Accordingly, various aspects which have yet to be explored in the literature have been identified. To start, although access to information regarding the management of these organizations is an essential aspect of effective banking CG, the issue of information transparency concerning the good governance of LA banking institutions remains an underdeveloped topic [11, 12]. In this regard, it should be noted that although there is a vast array of valuable literature on CG transparency in the context of non-financial institutions [13], the related findings do not lead to an understanding of the information transparency policies of financial or banking institutions since these organizations possess unique characteristics, including a) having many more stakeholders than other entities b) a high degree of leverage; c) are opaque, and d) belong to a heavily regulated sector [14, 15]. Therefore, there is a need for empirical studies that specifically look at access to information about CG practices in the banking context [16]. Moreover, stakeholders should receive this information regularly on an annual basis [17]. In this respect, longitudinal studies on banks’ transparency related to ethical management mainly focus on disclosing social responsibility practices [18], while those related to good governance disclosure remain scarce. Finally, in the main, the existing literature analyzes online dissemination of voluntary information regarding banks’ CG practices [19], with little attention being paid to the dissemination of mandatory information. Therefore, further and more in-depth research is needed on the extent to which the banking sector publishes both voluntary and mandatory information regarding its CG practices on its websites.

Given these precedents, this paper aims to contribute to the need for studies on transparency regarding CG in the banking sector, especially in emerging LA economies such as Paraguay. This country has not only been neglected in corporate governance literature but, as Paraguay is considered the second most corrupt country in LA, the issue of promoting information transparency is especially challenging [20]. Hence, this case study’s findings help enrich current literature on the extent to which banks that operate in highly corrupt countries make their CG practices visible. In particular, this paper aims to address the following research question: What factors influence online transparency information, both mandatory and voluntary, concerning the CG practices of Paraguayan banks? To this end, a longitudinal analysis was developed on the online transparency of Paraguayan banks from 2016 to 2019. In addition, a regression analysis based on an econometric data panel model was performed. This study seeks to provide consistent results on the influence of eight organizational factors on CG transparency practices in the Paraguayan banking sector.

The paper is organized as follows: the second section outlines a literature review on transparency in the banking sector, and the third section outlines the factors that determine CG disclosure. The fourth section explains the current study’s empirical methodology, including the model employed, followed by an analysis of the data and a discussion of the results. The last section presents the conclusions of this study, indicating managerial and policy implications of the findings.

## Transparency in the banking sector

In general, information access represents clear evidence of good CG practice in an organization [21–24]. The research in the banking sector highlights several benefits of transparency practice: a) making it easier for investors to evaluate more accurately the financial strength and performance of the bank; b) increasing the bank’s credibility; c) demonstrating the bank’s ability to manage risks; and, d) reducing uncertainty about its cash flow [16, 25].

Information transparency regarding board governance in banks is widely considered a guiding principle in CG best practice, as stakeholders demand to be informed about the Board’s proper management and processes to lower their levels of uncertainty regarding investment decisions. Therefore, among other aspects, it is essential for key bank stakeholders such as shareholders to monitor the board of directors’ performance [26].

Implementing suitable communication systems is essential for effective transparency practices [23]. In this regard, websites are considered vital sources of information for citizens. Banks should not only provide access to organizational information but also proactively disclose such information via their websites [26–28].

Most of the extant research regarding transparency in the banking sector is based on studies that mainly address access to the voluntary information of organizations in general (e.g., [10, 29, 30]). To a lesser extent, there are studies focused on specific aspects of banks’ management, such as social responsibility disclosure [18] or risk disclosure (e.g., [31, 32]). Nevertheless, despite the vital role corporate governance disclosure plays in businesses such as banks [33], most empirical studies on CG transparency have been developed under the context in the US publicly traded companies [16].

Concerning the literature explicitly addresses transparency on banking CG practices in emerging economies; the existing literature addressed cross-sectional and longitudinal studies in banks operating in Asian and African countries. Under a cross-sectional analysis, the outcomes indicate that Bangladesh banks’ overall CG transparency is high [34]. Similar results are noted in the Indian case, noting that mandatory disclosure is higher than voluntary disclosure [35]. Access to information regarding CG practices in banks operating in Ghana, Nigeria, and South Africa is low [19, 36]. Based on longitudinal analyses, a high level of disclosure regarding CG practices is found in banks operating in Iran, Saudi Arabia, Malaysia, [26], and Pakistan [37]. In contrast, in the Nigerian context, information transparency on banking governance is scarce [33]. In addition, previous research states that there is potential for improvement in the transparency policies observed in countries of the Arabian Gulf [38, 39] and Bangladesh [40].

Concerning the evolution of the transparency policies over time, although the level of CG information increased, it did not do so substantially. This outcome is observed in studies focused on a single country, such as in the case of Bangladesh [40] or Nigeria [33], and when addressing banks operating in several countries [12, 26, 33, 38, 39]. Regarding the content most frequently reported, in general terms, previous studies state the transparency related to board structure, audit committee, and financial information are the most prevalent. In contrast, the least frequently disclosed information is related to risk management and social responsibility practices [12, 19, 26, 35–38]. This research aims to bridge the literature gap on CG practices in emerging LA economies like Paraguay. It also aims to enrich current literature with greater knowledge on how transparency policies have evolved over time, with particular attention paid to the progress of voluntary and mandatory CG information disclosure of finance companies.

## Theoretical framework and development of hypothesis

Among the main theories that explain information disclosure policies for CG are Agency Theory [41] and Stakeholder Theory [42]. According to Agency Theory, the purpose of transparency is to reduce the asymmetries of information between managers and proprietors of banks. According to Stakeholder Theory, transparency targets a broader public as it aims to satisfy the information demands of other agents involved in the banking sector (e.g., clients, employees) [14]. Based on these theories, several hypotheses are presented.

### Size

For Jensen and Meckling [41], agency costs increase relative to company size. Consequently, the size of an organization has a considerable influence on stakeholders’ demand for transparency [8, 43, 44]. Concerning the banking sector, some studies evidence the positive influence of bank size on voluntary information disclosure associated with social responsibility practices (e.g. [18]) and financial information (e.g. [32]), albeit to a lesser extent, other studies did not identify a significant relationship [45]. Nevertheless, the aforementioned research is not specific to CG transparency; thus, more research on this issue is needed. Based on previous literature, it would be logical to expect that the Paraguayan banking sector would behave similarly to other types of entities. Therefore, the following hypothesis is formulated:

H1: Bank size positively affects the online information transparency, both voluntary and mandatory, regarding CG practices of Paraguayan banks.

### Profitability

In line with Agency Theory, when the entity enjoys times of bonanza, the agent might be more willing to be accountable to his principal, and, subsequently, the entity may be more interested in disclosing information to third parties [18]. Srairi and Douissa [29] show that banks’ profitability in emergent economies positively affects their transparency policies. Specific to information access to good governance practices, Rose [46] shows the positive relationship between CG transparency and the profitability of Danish companies. In LA, previous literature has not found a relationship between profitability and the level of compliance of practices related to good CG [47]. Based on the literature review and to further the understanding of the explanatory relationship between profitability and information transparency of CG practices of banks, the following hypothesis is formulated:

H2: Profitability positively affects the online information transparency, both voluntary and mandatory, regarding CG practices of Paraguayan Banks

### Leverage

In line with Agency Theory, companies with a high degree of leverage have many creditors and high agency costs due to the potential transfer of wealth from debtholders to shareholders, and consequently, their stakeholders demand a high level of information [48]. In previous studies focused on financial entities, a positive relationship between leverage and disclosure practices has been found [31, 32]. However, knowledge focused on the effect of leverage on CG disclosure in the banking sector is still scarce even though these are organizations with a high degree of leverage [15]. Considering that companies with greater leverage are more closely monitored by creditors, who press them to adopt good CG practices [49], this study aims to further understand this aspect with the following hypothesis:

H3: Leverage positively affects the online information transparency, both voluntary and mandatory, regarding CG practices of Paraguayan banks

### Liquidity

Based on Agency Theory, information transparency reduces the conflicts of interests between shareholders and creditors. Hence, companies with low liquidity disclose more information to justify their liquidity status vis-a-vis key stakeholders such as investors and creditors [50]. However, the results from previous studies are inconclusive and mainly on non-finance companies. In particular, previous studies, such as the work of Bhasin and Shaikh [51] based on the disclosure on the CG in Saudi Arabia, evidence a positive relationship between information disclosure on CG and high liquidity, whereas other studies, like the work of Lan et al. [50] conclude that liquidity is a non-significant factor in the voluntary information disclosure of Chinese companies. As liquidity risk affects how banks access cash in the short and long term [16], it is a key factor for the banking sector. Further research regarding to what extent liquidity is determinant for CG practices in financial entities is needed. Based on Agency Theory, the following hypothesis is formulated:

H4: Liquidity negatively affects the online information transparency, both voluntary and mandatory, regarding CG practices of Paraguayan banks

### Foreign ownership

According to Agency Theory, foreign investors tend to demand greater implementation of monitoring systems to oversee the management of local companies. Due to geographic and language barriers, foreign investors demand that directors provide a rich information environment to reduce the higher levels of information asymmetry they face compared to domestic investors [31]. Previous studies have posited that foreign ownership is a positive factor in good CG practices for banks [52]. Concerning transparency, previous studies confirm that foreign ownership positively affects access to corporate information [43, 53]. However, further specific research regarding the banking sector on the issue of CG is needed. Based on the literature review, the following hypothesis is formulated:

H5: Foreign ownership positively affects the online information transparency, both voluntary and mandatory, regarding CG practices of Paraguayan banks

### Credit risk rating

In line with Stakeholder Theory, banks as organizations are open systems that must satisfy the information needs of their stakeholders. One of these stakeholders, credit rating agencies, play a significant role in investment banking [14] as a bank’s risk rating is considered by other interested parties, among other aspects, as a good forecaster of a bank’s CG practices [54]. According to Bradford, Chen, and Zhao [55], banks’ transparency on CG practices positively influences the ratings received from these agencies. As a consequence of credit rating concerns, banking directors are interested in improving their CG practices [56]. Nevertheless, despite previous studies evidencing the impact of effective governance mechanisms on credit ratings [57], there is limited research on the influence of a bank’s credit risk on transparency practices. Hence, based on a literature review, this paper aims to advance this line of research with the following hypothesis:

H6: Credit risk ratings positively affect the online information transparency, both voluntary and mandatory, regarding CG practices of Paraguayan banks

### Audit firm size

In line with Agency Theory and Stakeholder theory, large audit firms can influence the level and quality of information disclosure regarding good CG practices [53]. The Law N° 861/96 of Financial Institutions [58] established that banks within the financial system must submit their Balance Sheet and Income Statement to independent external auditors. Literature has concluded that the quality of an audit has been associated with the four largest international audit firms, called the ‘Big Four.’ Therefore, it is expected that a client of a ‘Big Four’ firm would disclose a greater level of information [59]. In this regard, previous research confirmed the positive relationship between the size of the audit firm and the disclosure of CG information [60]. However scant is the attention to the context of the banking sector. According to the literature review, the following hypothesis is formulated:

H7: The size of the audit firm positively affects the online information transparency, both voluntary and mandatory, regarding CG practices of Paraguayan banks

### Listed on a stock exchange

Based on Agency Theory, corporations listed on a stock exchange have additional pressures and conflicts of interests among their stakeholders, and, consequently, information disclosure is fundamental for reducing their agency costs [33]. Previous studies have concluded that banks listed on a stock exchange disclose voluntary information in their Annual Reports to answer their stakeholders’ information needs [31]. Specific to CG transparency, the research into information access to banking CG practices is scarce. Therefore, more progress in this area of research is needed. Based on the literature review, the following hypothesis is formulated:

H8: Being listed on a stock exchange positively affects the online information transparency, both voluntary and mandatory, regarding CG practices of Paraguayan banks.

## Materials and methods

### Sample

The sample comprises all banks operating in the Paraguayan banking system for 2016–2019, consisting of 68 observations. The data was collected from the annual reports published and corporate governance data available on the websites of Paraguayan banks. In addition, secondary data was collected from the statistics published by the Superintendent of Banks of the Central Bank of Paraguay (CBP). In line with Isukul and Chizea [36] and Elmagrhi et al. [61], the sample period was limited to banks with data published on the Internet for consecutive years. This criterion helped us to satisfy the requirement of a balanced panel analysis. The 2019 financial year was the last year for which data was available at the time of data collection. Ergo, the following banks operating in Paraguay were analyzed: a) foreign direct branches: Citibank Paraguay N.A; Banco Do Brasil in Paraguay S.A, Banco de la Nación Argentina.; b) banks with majority foreign ownership: Banco Itaú Paraguay S.A., Sudameris Bank S.A.E.C.A., BBVA S.A., Banco GNB Paraguay S.A.; c) banks with majority local ownership: Banco Regional S.A.E.C.A., Banco Amambay S.A., Banco Continental S.A.E.C.A., Visión Banco S.A.E.C.A., Banco Rio S.A.E.C.A., Banco Familiar S.A.E.C.A., Banco Atlas S.A., Bancop S.A., Interfisa banco S.A.E.C.A. and c) banks with state ownership: Banco Nacional de Fomento.

### Descriptive analysis of online transparency

A content analysis was performed in line with previous studies regarding information disclosure in the banking sector (e.g. [12]). Content analysis was selected because of its reliability, validity, and the shortage of other means to effectively measure the extent of information disclosure. Unlike other methods, such as questionnaires or field studies, this analysis evaluates the information disclosure without the knowledge of the information communicator, making it a discrete analysis [44]. After an initial discussion, the coding was hand-coded by two coders working independently of each other, ensuring agreement in the scoring procedure [62].

The content analysis implemented is deductive as the information has been codified into pre-defined categories derived from the literature reviewed according to Berg [63]. An Index of Online Transparency of Corporate Governance (IOTCG, thereinafter) is developed, including mandatory and voluntary information. The items are based on the literature review and the regulatory framework of the banking system in Paraguay, in line with Resolution N° 65/2010 of the CBP [64] (see S1 Appendix). The first 15 items of the IOTCG correspond to mandatory information and analyzes the online information regarding the corporate rules related to the good governance of the bank, such as by-laws and shareholders meetings’ rules or other corporate rules such as Regulations of the Board of Directors [47, 64, 65] as well as information on the bank management such as corporate or Board Structure [12, 19, 28, 33, 38, 44, 64–66]. Likewise, the financial information available on financial statements from the last quarter and historical data are also included in the index according to similar studies and the regulation [28, 40, 64, 67, 68]. Moreover, previous research and regulation state the importance of information transparency concerning minutes of the shareholders’ meetings, dividend distribution policies, and shareholder agreements [43, 64]. The level of online access to annual reports and reports on corporate governance are also included [10, 39, 47, 64–66, 68].

The following 15 items relate to voluntary information disclosure of CG practices. In particular, information on the opinion of trustees and independent auditors, the annual report by the risk rating agency for current and previous years [28, 33, 65, 68–70]. Moreover, reports on the bank’s mission, vision, and principal values are also considered valuable in terms of voluntarily disclosed information [19, 44, 65]. In this vein, information on the ethical management of the entity based on a code of ethics, practices related to social and environmental responsibility are also considered essential to satisfy stakeholders’ information needs [23, 48, 67, 69]. Finally, online information about policies and procedures related to money laundering, goods awarded, and guarantees on deposited funds are also part of the IOTG [58, 70, 71, 72].The coding scheme employed was binary (unweighted) as it provides reliability and objectivity, may limit the possibility of bias towards any single or specific corporate governance provision, and is an approach used by a comprehensive and valuable range of literature, facilitating comparisons between the findings of this study with the results of past similar studies, in line with Elmagrhi et al. [61]. A score of “1” was assigned if the bank disclosed a particular item listed on the IOTCG on its website and “0” if it did not. The sum of the total number of items displayed on the Web is divided by the total number of items that should be disclosed. The disclosure for each bank is given in the following formula:

$${IOTGC}_{i}=\frac{{number\;of\;items\;bank}_{i}}{{{total\;possible\;number\;of\;items\;bank}_{i}}_{}}x\;100$$

### Econometric model

In line with previous studies on information disclosure, an econometric model of panel data has been developed [38]. This method overcomes the problem of bias from unobserved heterogeneity, which can affect cross-sectional models [73]. For Baltagi [74], there are various benefits to using this model, including controlling individual heterogeneity as it offers more information, more variability, less co-linearity between the variables, a greater degree of freedom, and greater freedom efficiency. The econometric model has a greater capacity to study the dynamics of adjustments.

In order to estimate the regression model of the data panel, a Hausman [75] specification test was carried out with a *null* hypothesis. The model of random effects is better than the fixed effects. For this hypothesis (p-value = 0.03939 < 0.05), fixed effects are preferred based on chisq = 13,239, df = 6; p-value = 0.03939. It was decided, therefore, to use fixed effects to attain a suitable model.

The dependent variable is the level of online information transparency, both mandatory and voluntary, of Paraguayan banks, according to IOTGC. Eight organizational factors were contrasted based on the hypotheses outlined (see Table 1) regarding the independent variables. The regression of the panel data comprised the years from 2016 to 2019 (τ = 4), and the resulting formula was:

IOTCGιτ=α+β1SIZE+β2ROAιτ+β3LEVιτ+β4LIQιτ+β5OWMιτ+β6CRRιτ+β7AUDιτ+β8AEXιτ+ε (1)

Where:

IOTCG = Index of Online Transparency of the Corporate Governance, ι = year, and τ = bank

SIZE = Size of the bank

ROA = Return on Assets

LEV = Leverage

LIQ = Liquidity

OWN = Foreign ownership

CRR = Credit Risk Rating

AUD = Size of the Audit Firm

LISTED = Listed on the ‘Bolsa de Valores y Productos de Asunción Sociedad Anónima’ (BVPASA) Stock Exchange, α = constant, and ε = error in forecasting.

**Table 1:** Independent variables.

Independent variables			Measurement	Authors	Expected sign
H1	Bank Size	SIZE	Natural Logarithm as of 31/12/2016, 2017, 2018, and 2019.	Sarea [28], Duc and Huong [43], Habbash et al. [44], Chakib [45], Gandía and Pérez [65], El-Halaby et al. [76]	(+)
H2	Return on Assets	ROA	Profitability on the total assets as of 31/12/2016, 2017, 2018, and 2019	Alali and Romero [8], Sarea [28], Munisi and Randoy [49], El-Halaby et al. [76],	(+)
H3	Leverage	LEV	Total Leverage as of 31/12/2016, 2017, 2018, and 2019.	Alali and Romero [8], Rodríguez and Noguera [48]	(+)
H4	Liquidity	LIQ	Current liquidity as of 31/12/2016, 2017, 2018, and 2019.	Albitar [77]	(-)
H5	Foreign ownership	OWN	Dichotomy variable with a value of 1 for direct foreign branch banks and with a majority of foreign participation and 0 for minority foreign participation.	Barako et al. [53], Duc and Huong [43]	(+)
H6	Risk Rating	CRR	Dichotomy variable with a value of 1 for the Rating AApy or higher and 0 for other ratings.	Kang and Ausloos [54], Bereskin et al. [56]	(+)
H7	Size of the Audit Firm	AUD	Dichotomy variable with a value of 1 for banks audited by ‘Big 4’ Firms and 0 for other audit firms.	El-Halaby et al. [76], Uyar et al. [59]	(+)
H8	Listed on the Stock Exchange, ‘Bolsa de Valores y Productos de Asunción Sociedad Anonima’ (BVPASA)	LISTED	Dichotomy variable with a value of 1 for Banks listed on the BVPASA Stock Exchange and 0 for the remainder.	Barako et al. [53] (2006), El-Halaby et al. [76]	(+)

## Results and discussion

### Descriptive results: CG transparency via websites

In general terms, more than half of the information regarding CG practices was disclosed by the banks that operate in Paraguay (see S1 Appendix). This “moderate” level of CG disclosure is in line with similar prior longitudinal research focused on access to information in the context of the banking sector in Arabian Gulf countries [38, 39] and Bangladesh [40]. However, this result does not concur with the high level of disclosure regarding CG practices found in banks operating in Iran, Saudi Arabia, and Malaysia [26] or the scant amount of research on banking governance in the Nigerian context [33]. Regarding the evolution of CG transparency practices, there is a slight variation over the four years analyzed, with only a slight increase identified in 2019. This finding is in line with previous studies in the banking sector, where a gradual increment in disclosure practice year on year is observed, albeit not in a significant manner [12, 33, 39].

There is a greater degree of voluntary information disclosure than mandatory information disclosure, approximately 10% more. This could be because disclosing additional information is considered a sound strategy to compete in the market and avoid additional regulations imposed on the sector.

Delving further into mandatory information transparency, the online information regarding the corporate rules related to the good governance of the bank, such as by-laws and shareholders meetings’ rules or rules of the Board, is low. However, most banks operating in Paraguay have made information about the people responsible for bank management, such as the corporate and board structure, more visible. Likewise, the financial information via financial statements both from the last quarter and historical data are also quite frequently reported on the websites of Paraguayan Banks. This high-level of online transparency about the organizational structure and financial information follows similar behavior displayed by banks operating in Asia [26, 37, 39] and African countries [19, 36].

In contrast, online information transparency concerning the minutes of the shareholders’ meetings, dividend distribution policies, and shareholder agreements is very low, albeit, in general terms, a positive evolution is observed over the years analyzed. Access to annual reports is provided in more than half of the Paraguayan banks, while the availability of reports on corporate governance is almost unanimous.

Concerning voluntary information disclosure, in 2016 and 2019, there was, on average, slightly more information available than in 2017 and 2018. This may be because, in general, Paraguay banks did not establish transparency policies in an online format until 2016. This “interest” in online transparency was neglected in the following two years until 2019, when an international organization, International Transparency, noted an increase in the level of corruption in Paraguay. Consequently, it seems that these entities increased their voluntary information disclosure on CG practices to legitimize the Board’s proper management and mitigate key stakeholders’ uncertainty regarding investment decisions in the banking system.

Concerning the contents of voluntary online transparency, information regarding the opinion of Trustees is less available than the opinion of independent auditors. Likewise, the interest in providing an annual report by the risk rating agency is high. These outcomes support previous studies that highlight the wide availability of information regarding the audit committee members [26] and the organization’s risk management [35, 38, 39].

More than half of the sample discloses information on the bank’s mission, vision, and principal values. However, the availability online of the banks’ code of ethics is limited, hence a lack of online information disclosure regarding how Paraguayan banks expect their employees to behave. These results are similar to those of previous studies conducted on Nigerian banks [33]. Moreover, since 2017, the percentage of Paraguayan banks that disclose social responsibility and sustainability information via their websites has decreased. This result agrees with previous studies from emerging economies [12, 19, 26, 35–38] but not with previous studies that noted a greater degree of information disclosure on such issues in US banking entities [78]. This behavior could be due to Paraguayan banks being more focused on financial information to justify the absence of corruption in the organization.

In 2017 and 2018, there was a drastic decrease in access to online information about policies and procedures related to money laundering. However, in 2019, there was a notable change towards greater disclosure on this issue. This increase in information disclosure in 2019 supports current studies highlighting the importance of banks’ response to emerging trends in money laundering and related crimes through improved transparency practices [79]. Finally, overall, half of the banks operating in Paraguay disclosed additional information on their websites regarding goods awarded and, to a lesser extent, guarantees on deposited funds.

### Results of the regression analysis

Table 2 reports the results of the fixed effect regression model. The fixed-effects model is jointly valid since the p-value (less than 2,373·10^(-11) related to the contrast of joint significance is less than 0.05. The Adjusted R-Squared of 0.6139 indicates that the forecasted variables of the model explain 61.39% of the variation in the IOTCG.

**Table 2:** Results of the regression analysis.

Estimate	Std.	Error	z-value	Pr(>|z|)	Expected Sign	Hypothesis
**(Intercept)**	0.499122	0.624280	0.800	0.427199		
**SIZE**	0.237938	0.051394	4.630	0.0000206***	+	Accepted
**ROA**	0.007195	0.010997	0.654	0.515467	+	Rejected
**LEV**	-1.416.651	0.560998	-2.525	0.014269*	+	Rejected
**LIQ**	-0.504092	0.154264	-3.268	0.001810**	-	Accepted
**OWN**	0.036762	0.038493	0.955	0.343462	+	Rejected
**CRR**	-0.173389	0.047299	-3.666	0.000530***	+	Rejected
**AUD**	-0.127419	0.039571	-3.220	0.002086**	+	Rejected
**LISTED**	0.160228	0.040065	3.999	0.000179***	+	Accepted

Signif. codes

*Significant by 10%

**Significant by 5%, and

***Significant by 1%.

Residual standard error: 0.1143 on 59 degrees of freedom.

Multiple R-squared: 0.66, Adjusted R-squared: 0.6139.

F-statistic: 14.32 on 8 and 59 DF p-value: 2.373e-11.

H1 is confirmed, indicating that bank size positively influences online disclosure of voluntary and mandatory information related to the CG practices of Paraguayan banks. This finding agrees with previous studies that examined the relationship between this variable and banking sector transparency practices focused on social responsibility [18] and financial issues [32]. In addition, this result supports previous studies focused on online transparency in non-financial entities [8].

H2 is not supported. The data indicated a neutral effect of the Paraguayan banks’ profitability on their transparency policy regarding CG. This outcome agrees with Briano and Rodriguez [47], who indicate no relationship between financial performance and the level of compliance related to good CG practices in companies in LA. However, the results do not concur with previous studies developed in other emergent countries that conclude that banks’ profitability positively affects the transparency policy of the organization [29]. Likewise, it does not coincide with previous research on CG transparency focused on non-finance companies [46].

Regarding H3, the data indicates that there is a significant but inverse relationship between leverage and IOTCG. In the case of Paraguayan banks, those entities with a high level of leverage are less keen to provide voluntary and mandatory information on their CG practices on their websites. This finding partially supports previous literature that indicated a significant but positive effect of this factor on information access to corporate information in the financial sector [31, 32].

The H4 is supported. The liquidity of banks is a determinant factor for IOTCG. The findings show that the relationship is indirect. Banks that operate in Paraguay with less liquidity are more interested in disclosing voluntary and mandatory information regarding their CG practices. This outcome does not coincide with previous research that states a direct relationship between liquidity and CG information disclosure, albeit, in this case, in the context of Saudi Arabia [27].

The H5 is not confirmed. In particular, the factor of “foreign ownership” shows a neutral effect on the IOTCG. It appears that Paraguayan banks are not very concerned about the information demands of foreign investors even though they experience manifestly greater information asymmetry than domestic investors [31]. In addition, this result does not support previous studies focused on information disclosure, albeit not specific to CG [43, 53].

The H6 results indicate a significant and inverse relationship between credit risk rating (CRR) and the IOTCG. This result does not confirm the expected sign in the working hypothesis, in that those banks with lower ratings and, thus, a higher credit risk are the most proactive in disclosing mandatory and voluntary information regarding their CG practices. According to Bradford, Chen, and Zhao [55] and Bereskin, Kim, and Dongchuhl [56], this result could be attributed to banks that operate in Paraguay with low credit ratings opting to implement transparency best practices as a strategy for improving their ratings. Moreover, the results support previous research emphasizing the relationship between risk rating and CG practices [54].

H7 is not confirmed since results indicate that the size of the audit firm has a significant but negative effect on online access to voluntary and mandatory information regarding the CG practices of Paraguayan banks. This outcome does not coincide with the results of previous research on voluntary corporate disclosure [59] and those specifically analyzing access to CG information [60], albeit not specific to the banking sector.

Finally, H8 is accepted. The results show that Paraguayan banks listed on a stock exchange provide greater online access to voluntary and mandatory information regarding their governance practices. This finding supports previous research that posits that publicly listed banks are more prone to respond proactively to the information needs of their stakeholders [31] as they are subject to additional pressures and more conflicts of interest among their stakeholders.

## Conclusions

This study aims to advance the literature regarding online access to banks’ corporate governance information in emerging economies from Latin America. In particular, it addresses the following research question: What are the influencing factors in the online transparency of mandatory and voluntary information related to the CG practices of Paraguayan banks? To this end, a longitudinal and descriptive analysis was developed concerning online information access to the CG practices of Paraguayan banks from 2016 to 2019. In addition, an empirical analysis via a regression analysis using a data panel econometric model was performed.

Based on the findings of the descriptive analysis, it can be concluded that banks that operate in Paraguay present a moderate level of online disclosure regarding their CG practices on their websites. The evolution of the information disclosure regarding banks’ governance has made little “progress” over the years analyzed. In addition, information disclosure for each of the CG practices analyzed is heterogeneous. Surprisingly, Paraguayan banks do not appear interested in disseminating information online about their compliance with mandatory laws and regulations related to their CG practices. Instead, they provide greater online information transparency of voluntary information. Hence, while previous studies call for banks in emerging Asian economies such as Bangladesh [40] and the Arab Gulf [38, 39] and African countries such as Nigeria [33] and South Africa [36], to increase their corporate disclosure, this study concludes that, in LA countries such as Paraguay, more extraordinary efforts are needed to reduce the information asymmetry between these financial entities and their stakeholders.

The findings of the empirical analysis concluded that, in line with Agency Theory and Stakeholder Theory, the organizational factors of “size,” “leverage,” “liquidity,” “credit risk rating,” “size of the audit firm,” and “listed on a stock exchange” have a statistically significant relationship with the information transparency regarding CG practices of the Paraguayan banking sector. Thereby, this paper adds to the current literature about emerging economies and determinant factors in the transparency of CG (e.g. [19, 22]). It also notes the importance of these theories for understanding transparency practices in the LA context and, more specifically, in the case of the Paraguayan banking sector. In particular, it confirms that large banks, subject to a high degree of public scrutiny, are more proactive in providing information on their websites regarding how the management board is structured. In addition, those entities with a high level of leverage, and thus with many creditors, appear not to be interested in reducing their agency costs by responding to the information needs of their key stakeholders.

Moreover, Paraguayan banks with less liquidity and higher credit risk are more interested in using web transparency to justify their management to key stakeholders such as investors, creditors, and credit rating agencies. Finally, being audited by one of the ‘Big 4’ audit firms does not encourage Paraguayan banks to improve the visibility of their good governance in online environments. In contrast, banks listed on a stock exchange show greater concern for CG transparency.

Pertaining to managerial implications, management boards are responsible for fostering information transparency about the entity’s culture. This aspect has been truly neglected by all banks that operate in the Paraguayan banking system since the level of CG transparency is, on average, moderate. This neglect should be urgently addressed as greater transparency about the country’s banking system’s practices could make investing in Paraguay’s banking system more attractive. Directors of banks that operate in Paraguay should bear in mind that online transparency on CG practices is a strategic practice to mitigate the uncertainty felt by potential stakeholders’ about banking management, given the importance of this aspect considering the high levels of corruption in the country. To this end, the “CG mandatory information” should be made more visible to the public. In addition, concerning “CG voluntary information,” there is significant room for improvement in disclosure practices of these banks related to social and environmental responsibility. Nowadays, and considering the current COVID-19 pandemic, banks should prioritize corporate sustainability issues and be transparent about such practices. In addition, the sector itself could also develop self-regulation mechanisms that encourage the Boards of banks to report on their governance practices not just for facilitating stakeholder oversight of the financial institutions’ management but also as a mechanism for continual improvement. Regarding the use of the Internet, banks should be aware of the importance of exploiting “the capacity of online platforms” such as websites and social networking sites to foster stakeholders’ interest, such as clients, on how the bank is run, this stakeholder engagement via online platforms can be a strategic resource to foster true confidence in the banking sector.

Regarding the main policy implications, the results of this study call for greater reflection on the actions that the relevant authorities could develop. One such recommended action would be the development of a manual detailing the CG practices that should be reported and proactively disclosed on the Internet. Compliance with such reporting guidelines within the Manual should involve all the organizations within the banking system, independently of their organizational factors. One of the authorities that could take up this challenge in Paraguay is the Paraguayan Central Bank. This institution should compel banks to abide by the rules of transparency and bolster the importance of disseminating data of interest to investors, clients, researchers, and other parties.

Several research areas related to online transparency on CG practices in the banking sector are yet to be studied. First of all, the research described in this paper is based on quantitative and longitudinal analyses with a limited sample of banks that operate in the Paraguayan banking system. It would be helpful to undertake further research using qualitative methodologies that deepen understanding of the transparency policies of the sector and studies that could address comparable situations in other geographical areas. Moreover, the online transparency analysis is expressed in terms of information available via websites. However, we are a network society, and social networking sites are daily communication mechanisms, so; it would be useful to understand how these online platforms fit into the communication strategies of LA banks.

## Supporting information

S1 Appendix Index of online transparency of corporate governance. (DOCX)
