# Supplementary material for: What determines the progress of online information access to banking corporate governance practices? The case of Paraguay
Source: PLoS One. 2022 Jan 5;17(1):e0262334. doi: 10.1371/journal.pone.0262334 (PMC8730433; doi:10.1371/journal.pone.0262334)
Supplement: S1 Appendix — (DOCX) [file pone.0262334.s001.docx]

|  |  |  |  | **VALUES** | | | |
| --- | --- | --- | --- | --- | --- | --- | --- |
|  |  |  |  | **2016** | **2017** | **2018** | **2019** |
| **Total** | | | | **57%** | **56%** | **56%** | **61%** |
| ***Total Online transparency: mandatory information*** | | | | 51% | 51% | 51% | 56% |
| ***Total Online transparency: voluntary information*** | | | | 63% | 61% | 61% | 65% |
|  |  |  |  |  |  |  |  |
|  |  | **Items** | **Literacy Support** |  |  |  |  |
| ***Online transparency: mandatory information*** | **1** | By-laws | *Briano and Rodríguez* [47]*, CBP* [64]*, Gandía and Pérez* [65] | 29% | 29% | 29% | 47% |
|  | **2** | Shareholders’ Meeting Rules | *CBP* [64] | 12% | 12% | 12% | 18% |
|  | **3** | Other Corporate Rules (Rules of the Board) | *CBP* [64] | 24% | 24% | 24% | 29% |
|  | **4** | Corporate Structure and Organization | *Sencal and Asutay* [12], *Isukul and Chizea* [19]*, Sarea* [28], *Herbert and Agwor* [33], *Habbash et al.* [44], *CBP* [64]*, BIS* [66]*, Srairi* [38] | 94% | 88% | 88% | 94% |
|  | **5** | Board Structure and Organization | *Sencal and Asutay* [12]*, Herbert and Agwor*[33], *Briano and Rodríguez* [47]*, CBP* [64]*,* *BIS* [66]*, UNCTAD* [67]*, BCRA* [68] | 94% | 88% | 88% | 88% |
|  | **6** | Financial Statements for the last full quarter | *Sarea* [28]*, CBP* [64] | 88% | 82% | 82% | 94% |
|  | **7** | Historical Financial Statements for the current year | *Sarea* [28]*, Srairi* [38], *CBP* [64]*,* *UNCTAD* [67]*,* *BCRA* [68] | 88% | 82% | 82% | 94% |
|  | **8** | Historical Financial Statements for the previous year | *Sarea* [28]*, Srairi* [38], *CBP* [64]*,* *UNCTAD* [67]*,* *BCRA* [68] | 82% | 88% | 88% | 94% |
|  | **9** | Minutes of the Shareholders’ Meetings for the current year | *CBP* [64] | 6% | 24% | 24% | 18% |
|  | **10** | Minutes of the Shareholders’ Meetings for the previous year | *CBP* [64] | 6% | 24% | 24% | 18% |
|  | **11** | Dividends Policy | *CBP* [64]*,* *Duc and Huong* [64] | 6% | 6% | 6% | 6% |
|  | **12** | Shareholders Agreements | *CBP* [64] | 6% | 12% | 12% | 6% |
|  | **13** | Annual Report by the Board for the current year | *Sarea* [28], *Srairi* [38]*, Habbash et al.* [44], *Chakib* [45]*, Briano and Rodríguez* [47]*, CBP* [64]*, BCRA* [68] | 65% | 59% | 59% | 65% |
|  | **14** | Annual Report by the Board for the previous year | *Sarea* [28], *Briano and Rodríguez* [47], *CBP* [64]*, BCRA* [68] | 65% | 71% | 71% | 76% |
|  | **15** | Report on the Corporate Governance | *Forte et al.* [10]*, Srairi* [38]*, Briano and Rodríguez* [47]*, CBP* [64]*, Gandía and Pérez* [65], *BIS* [66]*, BCRA* [68] | 100% | 82% | 82% | 100% |

S1 Appendix: Index of Online Transparency of Corporate Governance

| ***Online transparency: voluntary information*** | **16** | Opinion of the Trustee for the current year | *BCRA* [68] | 47% | 29% | 29% | 41% |
| --- | --- | --- | --- | --- | --- | --- | --- |
|  | **17** | Opinion of the Trustee for the previous year | *BCRA* [68] | 47% | 41% | 41% | 41% |
|  | **18** | Opinion of the Independent Auditor for the current year | *Sarea* [28], *Hossain* [35]*, Srairi* [39], *Gandía y Pérez* [65]*, BCRA* [68] | 76% | 76% | 76% | 94% |
|  | **19** | Opinion of the Independent Auditor for the previous year | *Sarea* [28], *Hossain* [35]*, Srairi* [39], *Gandía y Pérez* [65]*, BCRA* [68] | 76% | 88% | 88% | 94% |
|  | **20** | Annual Report by the Risk Rating Agency for the current year | *Sareen and Vij* [69]*, Kang and Ausloos* [54]*, Srairi* [38] | 76% | 82% | 82% | 88% |
|  | **21** | Annual Report by the Risk Rating Agency for the previous year | *Sareen and Vij* [69]*, Kang and Ausloos* [54] | 76% | 88% | 88% | 88% |
|  | **22** | Vision Statement, Mission Statement and Corporate Values | *Isukul and Chizea* [19], *Habbash et al.* [44], *Gandía y Pérez* [65] | 65% | 53% | 53% | 65% |
|  | **23** | Information on the main management | *Hossain* [35]*, Srairi* [38], *Briano and Rodríguez* [47], *BCRA* [68] | 94% | 76% | 76% | 82% |
|  | **24** | Code of Ethics | *Sarea* [28], *Briano and Rodríguez* [47], *Gandía y Pérez* [65]*, UNCTAD* [67]*, BCRA* [68] | 12% | 18% | 18% | 18% |
|  | **25** | Social Responsibility Information | *Forte et al.* [10], *Sarea* [28], *Briano and Rodríguez* [47], *Gandía y Pérez* [65]*, UNCTAD* [67] | 71% | 59% | 59% | 59% |
|  | **26** | Environmental Responsibility Information | *Forte et al.* [10], *Sarea* [28], *Briano and Rodríguez* [47],  *UNCTAD* [67] | 47% | 41% | 41% | 41% |
|  | **27** | Information on Duties and Tariffs | *CBP* [70] | 100% | 100% | 100% | 100% |
|  | **28** | Information on Policies and Procedures for the Prevention of Money Laundering (PLD) | *Law N° 1015/97*[71] | 71% | 47% | 47% | 82% |
|  | **29** | Information on goods awarded | *Law N° 861/96*[58] | 47% | 65% | 65% | 53% |
|  | **30** | Information on the Fund of Deposits Guarantee | *Law N° 2334/03*[72] | 35% | 47% | 47% | 35% |
